# ﻿Rediscovery of *Gasteranthusextinctus* L.E.Skog & L.P.Kvist (Gesneriaceae) at multiple sites in western Ecuador

**DOI:** 10.3897/phytokeys.194.79638

**Published:** 2022-04-15

**Authors:** Nigel C. A. Pitman, Dawson M. White, Juan Ernesto Guevara Andino, Thomas L. P. Couvreur, Riley P. Fortier, José Nicolás Zapata, Xavier Cornejo, John L. Clark, Kenneth J. Feeley, Mark K. Johnston, Alix Lozinguez, Gonzalo Rivas-Torres

**Affiliations:** 1 Keller Science Action Center, Science and Education, Field Museum of Natural History, 1400 S. Du Sable Lake Shore Dr., Chicago, IL 60605 USA; 2 Negaunee Integrative Research Center, Science and Education, Field Museum of Natural History, 1400 S. Du Sable Lake Shore Dr., Chicago, IL 60605 USA; 3 Grupo de Investigación en Biodiversidad, Medio Ambiente y Salud-BIOMAS, Universidad de las Américas, Campus Queri, Quito, Ecuador; 4 DIADE, Univ Montpellier, CIRAD, IRD, Montpellier, France; 5 Pontificia Universidad Católica del Ecuador, Facultad de Ciencias Exactas y Naturales, Av. 12 de Octubre 1076 y Roca, Apartado 17-01-2184, Quito, Ecuador; 6 Department of Biology, University of Miami, 1301 Memorial Drive, Coral Gables, FL 33146, USA; 7 Herbario QCA, Escuela de Ciencias Biológicas, Pontificia Universidad Católica de Ecuador, Av. 12 de octubre 1076 y Roca, Apartado 17-01-2184, Quito, Ecuador; 8 Herbario GUAY, Departamento de Botánica, Facultad de Ciencias Naturales, Universidad de Guayaquil, Av. Raúl Gómez Lince s.n. y Av. Juan Tanca Marengo (campus Mapasingue), Apartado 09-01-10634, Guayaquil, Ecuador; 9 Science Department, The Lawrenceville School, Lawrenceville, NJ 08648, USA; 10 Estación de Biodiversidad Tiputini, Colegio de Ciencias Biológicas y Ambientales, Universidad San Francisco de Quito USFQ, Quito, Ecuador

**Keywords:** Andes, Centinela, Chocó, cloud forest, endemic, extinction, iNaturalist, tropical forest

## Abstract

We report the rediscovery of the Critically Endangered cloud forest herb *Gasteranthusextinctus*, not seen since 1985. In 2019 and 2021, *G.extinctus* was recorded at five sites in the western foothills of the Ecuadorian Andes, 4–25 km from the type locality at the celebrated Centinela ridge. We describe the species’ distribution, abundance, habitat and conservation status and offer recommendations for further research and conservation efforts focused on *G.extinctus* and the small, disjunct forest remnants it occupies.

## ﻿Introduction

**Figure 1.**
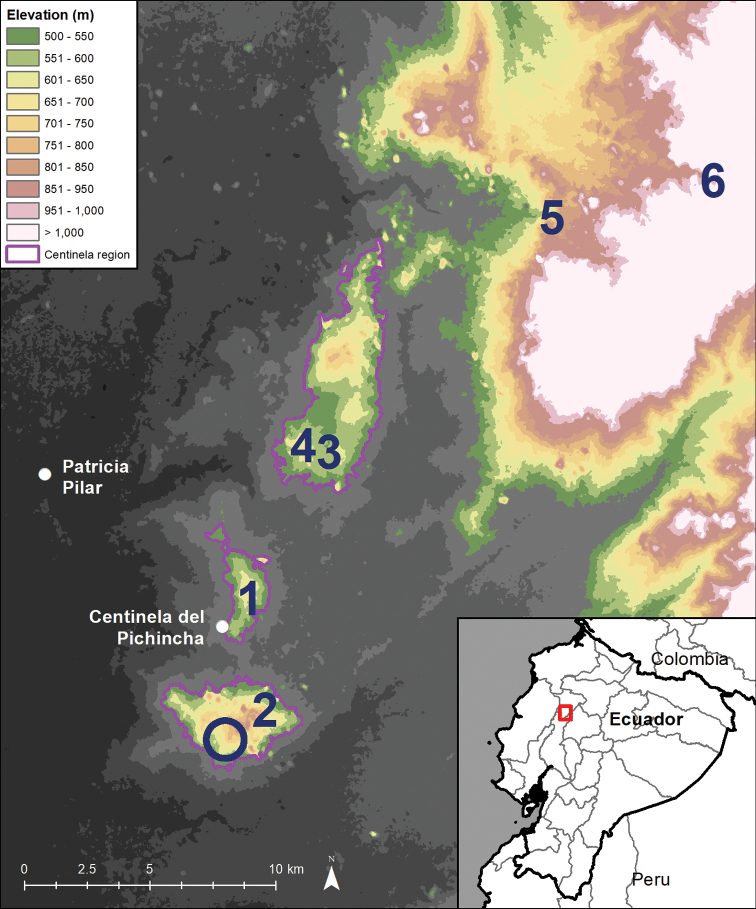
Map of the Centinela Region and documented localities of *Gasteranthusextinctus*. Numbers indicate the approximate locations of all populations confirmed to date, including the original collection locality (1) and the four sites where we observed the species in 2021 (2–5). Site 6 is from iNaturalist occurrence records. The hollow circle indicates a site where we searched for, but did not find *G.extinctus*. See Appendix 1 for a detailed description of each site. Inset: the placement of the Centinela Region in the Santo Domingo de los Tsáchilas Province (grey lines) in western Ecuador.

Extensive deforestation in western Ecuador during the 20^th^ century resulted in an alarming loss of habitat and the presumed extinction of a number of plant species with small geographic ranges (Dodson and Gentry 1991). *Gasteranthusextinctus* L.E.Skog & L.P.Kvist (Gesneriaceae) is a low terrestrial herb with uniformly bright orange flowers (Skog and Kvist 2000) and one of the 26 species of the genus currently known to occur in western Ecuador (Kvist et al. 2004). At the time of its description in 2000 (Skog and Kvist 2000), the only known records were four collections made between 1977 and 1985 in cloud forests at Centinela (Fig. 1 and Appendix 1, site 1), “an Andean foothill ridge… isolated from the main Andean range farther east by a broad, flat valley about 15 km wide” (Dodson and Gentry 1991: 277). Visited repeatedly by plant collectors in the 1970s and 1980s, Centinela became a celebrated site because of the dozens of apparently undescribed and endemic species in its flora (Gentry 1986a, 1986b, 1989; Gentry and Dodson 1987; Dodson and Gentry 1991; Dodson and Gentry 1993). These same publications reported that Centinela’s forests had been completely destroyed and converted to farmland by the year 1990 and posited that a large number of its putative endemics had become extinct.

This hypothesis was amplified by E. O. Wilson’s (1992) coining of the phrase ‘Centinelan extinction’ to describe geographically localised species that are driven to extinction by habitat destruction before they can be discovered or described. These reports prompted Skog and Kvist (2000) to give *G.extinctus* its dramatic specific epithet. They noted in the species description that “all four collections come from… [a] forest [that] has been totally cleared, likely causing the extinction of this species” (Skog and Kvist 2000: 67).

Around the time of the description, however, scientists began reporting that a large number of plant species once thought endemic to Centinela had been found at other sites (Pitman et al. 2000). Four years after describing *G.extinctus*, Kvist et al. (2004) themselves noted that five of the six *Gasteranthus* species considered Centinela endemics by Dodson and Gentry (1991) had been found elsewhere, leaving *G.extinctus* as the only remaining Gesneriaceae believed to be endemic to Centinela. During the same period, botanists who visited Centinela reported that small stands of forest still remained in the region (e.g. W. Alverson, pers. comm. to N. Pitman). Together, these lines of evidence supported the competing hypothesis that *G.extinctus* was potentially still extant, both at Centinela and elsewhere (Scheffers et al. 2011; Watson and Davis 2017).

At least one previous targeted search failed to locate new populations. For three days in 2009, J. L. Clark searched a lowland site 7 km WNW of Centinela (the Río Palenque Science Center) and surrounding areas for *G.extinctus*. That search did not locate any extant forest fragments outside of Río Palenque.

## ﻿Methods

In June-November 2021, we searched for *Gasteranthusextinctus* in three large Ecuadorian herbaria (QCNE, QCA, GUAY) and in GBIF (https://www.gbif.org) data from Ecuador, Colombia and Peru (DOIs: https://doi.org/10.15468/dl.x7j8cj, https://doi.org/10.15468/dl.3anwv6 and https://doi.org/10.15468/dl.ajrxp3, respectively). Those searches revealed no records beyond those mentioned in the species protologue.

**Figure 2.**
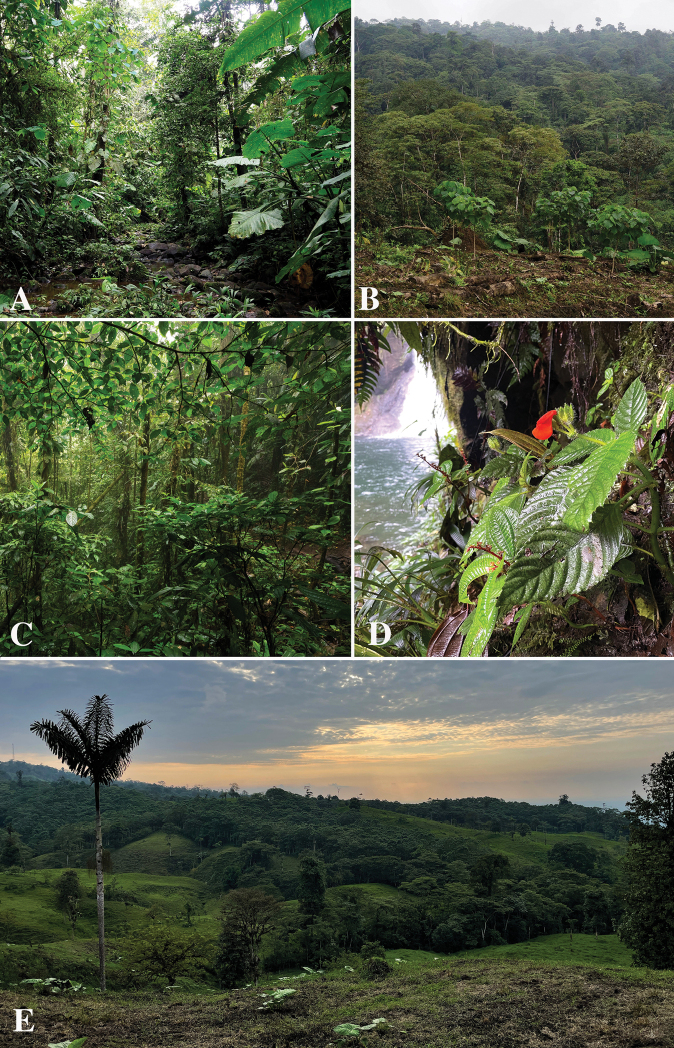
Field photographs of *Gasteranthusextinctus* habitats in the Centinela Region of western Ecuador **A** small stream where *Thomas Couvreur et al. 1502* was collected **B** recently planted *Gmelinaarborea* plantation and forest **C** cloud forest understorey **D***D. White et al. 830* river-side at Bosque y Cascadas Las Rocas **E** steep hills covered in a mosaic of cattle pasture, tree plantations and forest remnants. Photographs **A, B** by T.L.P. Couvreur **C** by R. Fortier **D** by D. White **E** by N. Pitman.

On 13–15 November 2021, we visited the Centinela Region to search for *G.extinctus* and other putative Centinela endemics (see Appendix 1 for notes on geographic names). Over three days of fieldwork, our 10-person team travelled the extensive network of rural roads by truck, searching for remnants of primary forest above 500 m. We observed > 20 such remnants (Fig. 2), most of them strips of forest along ravines or small (< 5 ha) patches on slopes too steep for the most common land uses in the region: dairy farming or plantations (mostly banana, balsa, *Gmelinaarborea* and cacao). We also confirmed the existence of one remnant of well-preserved forest measuring at least 50 ha and large enough to maintain a population of the Ecuadorian mantled howler monkey (*Alouattapalliataaequatorialis* [Festa, 1903]). We were told another large remnant with howler monkeys exists in the northern part of the Centinela Region, south of Bellavista. These landscape observations will be reported elsewhere in greater detail.

## ﻿Results and discussion

**Figure 3.**
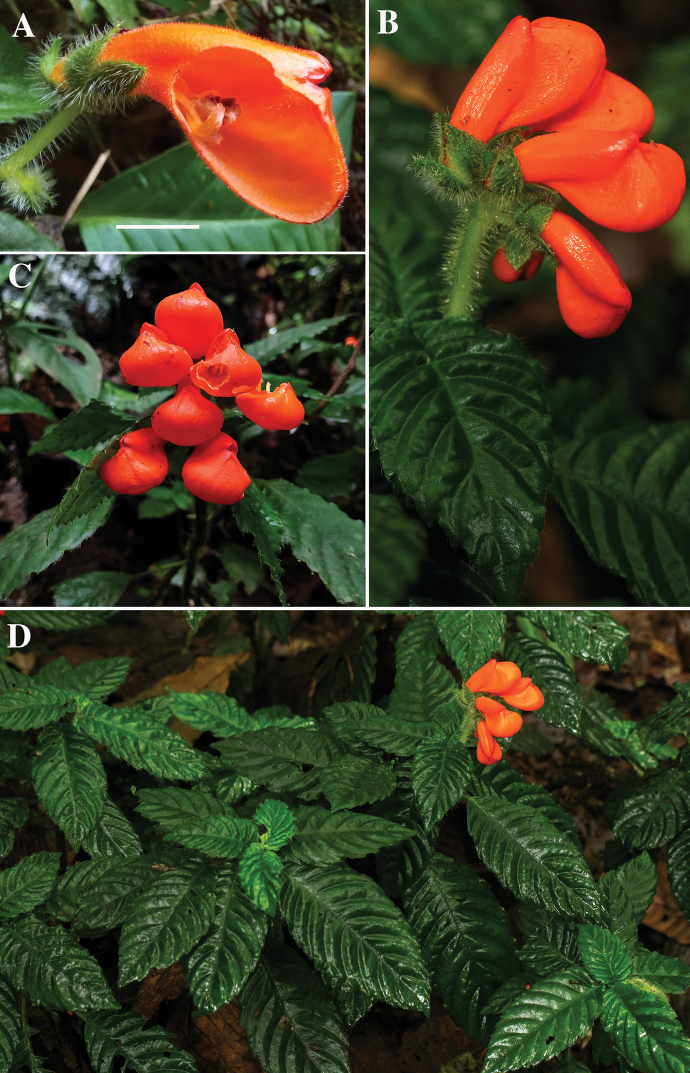
Field images of *Gasteranthusextinctus***A** herbivore-damaged corolla showing inflorescence branches and calyx with conspicuous pilose-villous indument **B** relatively (< 4 cm) short peduncles **C** relatively fewer flowers (2–6) per inflorescence and pouched or hypocyrtoid corollas **D** elliptic leaves. Photographs **A** by X. Cornejo **B, D** by R. Fortier **C** by N. Zapata. Scale bar: 1 cm.

We spent 2–6 hours searching each of four remnant patches of forest in the Centinela Region and encountered *G.extinctus* (Fig. 3) at three of them (Fig. 1 and Appendix 1, sites 2–4). During the same dates, we also recorded *G.extinctus* at one site close to, but outside of, the Centinela Region, on the main flanks of the Andes (Fig. 1 and Appendix 1, site 5).

Identification of the plants was straightforward. All five diagnostic characters mentioned in the original description were evident in the observed individuals (Fig. 3): “1) urceolate [with a protruding pouch], relatively large corollas (2.8–4 cm long); 2) inflorescences with relatively short peduncles (maximally 4 cm long); 3) few flowers (2–4) per cyme; 4) a conspicuous pilose-villous indumentum on stems, inflorescences and calyces; and 5) fairly small, elliptic leaves (maximally 11 cm long)” (Skog and Kvist 2000: 67). Plants observed in the field closely resembled the original line drawing (Skog and Kvist 2000: fig. 26). The two *Gasteranthus* species mentioned by Skog and Kvist (2000) as most closely resembling *G.extinctus* (*G.calcaratus* Kunth (Wieler) and *G.mutabilis* L.E.Skog & L.P.Kvist) were easily eliminated as possibilities, due to our plants’ conspicuous indumentum and pouched (or hypocyrtoid) urceolate corollas, respectively.

Field images of the plants were immediately shared with J.L. Clark, who was not part of the field team, but who is a taxonomic expert of Gesneriaceae with extensive knowledge of the flora of western Ecuador. By comparing the field images with the species description, an image of the holotype specimen (*C.H. Dodson 11595*, QCNE) and a field image taken of the species by C. Dodson in the 1980s, he confirmed that the plants were *G.extinctus*.

These populations were vouchered via five herbarium specimens under five different collector series (Appendix 1). The majority of these sheets will be deposited at four Ecuadorian herbaria (GUAY, QCA, QCNE and QUSF); duplicates will be deposited at herbaria outside of Ecuador (likely F, SEL, US, WAG and P). For all specimens, 1–3 leaves were stored in silica gel desiccant for genetic analysis; these were deposited at QUSF. Permits for herbarium voucher and DNA collection are listed in the acknowledgements section of this paper.

Field photographs will be linked with herbarium specimen databases and made available on GBIF. We have also posted three field photographs of *G.extinctus* on the community science platform iNaturalist (https://www.inaturalist.org; Appendix 1).

### ﻿Additional records

After completing our fieldwork, we observed on iNaturalist three records predating our field work that showed flowering plants we recognised as *G.extinctus*. Identified as *Gasteranthus* sp., the three records were made on a single day (30 November 2019) by three iNaturalist users and two show the same plant. We were not able to determine the precise locality or localities of these records from the iNaturalist records alone. We contacted the users, three university students at Ecuador’s Armed Forces University (ESPE) and learned that all three records were made at the Cascadas de Cristal Private Conservation Area near our site 5 (Fig. 1 and Appendix 1, site 6).

### ﻿Habitat, abundance and phenology

*Gasteranthusextinctus* was neither rare nor common at the sites where we observed it. It was, however, conspicuous due to its large and brightly coloured orange flowers and relatively easy to find. At two of the sites where it occurred, we sighted *G.extinctus* within the first 10 minutes of exploration. In some places, the species occurred as isolated individuals and in others as small clumps (i.e. 10 individuals in an area of 4 m^2^). Nowhere we visited was *G.extinctus* the dominant species in the understorey, but at some sites (and at some smaller areas within the sites), it appeared to be the most common terrestrial Gesneriaceae.

The populations of *G.extinctus* which we observed suggest a very broad environmental tolerance. We found individuals inside well-preserved forest and in cattle pastures just outside of forest; in deeply shaded understorey and in sunny open conditions; on soil with few to no rocks, on soil mixed with small rocks and on vertical rock walls near waterfalls; close to streams and far from them; and from 520 to 990 m elevation. The most commonly observed habitats were steep to vertical walls of damp soil along ravines, within 10 m of streams or rivers, inside relatively well-preserved forest (Fig. 2A–D).

In the places *G.extinctus* occurred, it was accompanied by a number of terrestrial aroids, ferns (*Diplazium*, *Danaea*, *Asplenium*, *Dennstaedtia*, *Tectaria*) and other Gesneriaceae (most conspicuously *Gasteranthuscorallinus* (Fritsch) Wiehler). In habitats on steeper slopes, some of the common and conspicuous trees we recorded around *G.extinctus* populations were *Carapamegistocarpa* A.H. Gentry & Dodson, *Talisiaequatoriensis* Acev.-Rodr., *Faramea* sp., *Quararibea* sp., *Swartziadecidua* Torke & Á.J.Pérez, *Eschweilerarimbachii* Standl., *Eschweileraawaensis* S.A.Mori & Cornejo, *Browneopsismacrofoliolata* Klitg., *Socratearostrata* Burret and *Wettiniaquinaria* (O.F.Cooke & Doyle) Burret. In habitats on less rugged topography, other conspicuous woody elements included *E.rimbachii*, *Carpotrocheplatyptera* Pittier, *Bauhiniapichinchensis* Wunderlin and numerous species in the genera *Inga*, *Matisia*, *Faramea* and *Jacaratia*. Common epiphytes included the orchids *Sobraliavalida* Rolfe, *Platysteleacutilingua* Kapuler & Hascall, *Scaphosepalum* sp., *Lepanthes* sp. and the bromeliads *Guzmaniawittmackii* (André) André ex Mez and *Guzmaniarhonhofiana* Harms. Field photographs of several other plant species that co-occur with *G.extinctus* at Centinela are accessible at https://www.inaturalist.org/projects/flora-of-centinela.

The original description notes that flowering specimens were collected in July, August and October (Skog and Kvist 2000). All new records reported here were flowering in November. We did not observe fruits, whose phenology and specific description remain unknown to science, but which are presumed to be laterally compressed semi-fleshy capsules like all other *Gasteranthus*.

### ﻿Conservation status

*Gasteranthusextinctus* is currently considered Critically Endangered, both globally (Clark et al. 2004) and in Ecuador (León-Yánez et al. 2011). The newly-discovered populations necessitate a reassessment of the species’ threat status. We analysed our six unique occurrences with the R package ConR (Dauby et al. 2017) and identified two subpopulations and four locations, based on a 10-km radius for equal impact. We estimated an area of occupancy (AOO) of 106 km^2^ and an extent of occurrence (EOO) of 20 km^2^ (grid resolution = 2 km). However, the species’ habitat is severely fragmented within this area. These values would place *G.extinctus* in the Endangered (EN) category (AOO < 500 km^2^, EOO < 5000 km^2^, locations < 5). The massive scale of the habitat loss since its discovery and the lack of formal protection means that the ‘B’ criterion applies, resulting in a new proposed assessment of EN B1(a,b(ii,iii,iv)) +B2(a,b(ii,iii,iv)).

However, our field observations offer a measure of optimism regarding the plant’s conservation prospects. Most importantly, it appears that significant populations may occur within private conservation areas (Appendix 1, sites 5–6). Others could potentially occur in the Murocomba Protection Forest; this requires confirmation. The species’ broad habitat tolerance, preference for ravines and ability to grow on sheer rock walls mean that there is a relatively large amount of high-quality habitat in the Centinela Region and on the nearby flanks of the Andes that is unlikely to disappear even with continued deforestation.

We did not collect live specimens of *G.extinctus*. Given its broad environmental tolerances, however, the species appears to be an excellent candidate for *ex situ* conservation. Observations of other streamside species of Gesneriaceae, native to western Ecuador (Ertelt 2013), suggest that *G.extinctus* likely possesses root-shoot vegetative propagation and might be easily propagated *ex situ*. However, the plant’s striking appearance also puts it at risk of unsustainable harvesting and trafficking of live specimens by commercial plant collectors (Lavorgna et al. 2018).

## ﻿Conclusion

A short burst of targeted fieldwork demonstrated that *Gasteranthusextinctus*, long considered both extinct and endemic to the Centinela Region, is in fact neither. The ease with which it was located at four sites in three days suggest that the species has a larger population and geographic distribution than previously recognised. Likewise, its broad tolerance of environmental conditions suggests relatively high frequency even in a massively modified landscape like this one. This implies a global population size in the thousands, at least several dozen individuals of which would appear to grow inside a formally-protected area. These field observations suggest that *G.extinctus*, while still meriting globally Endangered status, might be capable of persisting *in situ* even if the largest forest fragments in the region are not conserved.

Our work with *G.extinctus* underlines the urgency of targeted fieldwork to assess the conservation status of the dwindling forest fragments throughout western Ecuador and of the range-restricted plant species that depend on them. On the research front, what is needed is a comprehensive update of Dodson and Gentry’s (1991) survey of biological extinction in western Ecuador, backed by satellite imagery analysis, field surveys of remnant forests and field, herbarium and modelling work to understand the current status and distribution of endemic species. That research should also include newly-available tools to characterise extinction risk and effective population size, such as metabolomic analyses (Wetzel and Whitehead 2020) and population genomics (Wang et al. 2016). We also call on researchers to update Ecuador’s *Red List of Endemic Plants*, a vital resource for Ecuador’s large endemic flora, last published a decade ago (Valencia et al. 2000; León-Yánez et al. 2011).

On the conservation front, it is now clear that published reports of the total destruction of Centinela’s world-famous cloud forests were premature. Significant remnants of relatively healthy, intact forest persist in the Centinela Region. None of these remnants is formally protected and all of them are vulnerable to conversion to pasture or plantations in the near future. Especially in the southernmost, highest-elevation portions of the Centinela Range, a concerted campaign of land protection and habitat restoration has the potential to protect a contiguous, > 100-ha block of Centinelan cloud forest. Success will require coordinated efforts by local landowners, Ecuadorian government agencies, conservation NGOs and other stakeholders to ensure the long-term persistence of these remnants and the *G.extinctus* populations they harbour.

## ﻿Appendix 1

**A list of sites with documented occurrences of *G.extinctus* and notes on toponymy at the study site in western Ecuador. For each site, we provide a description of the locality, notes on *G.extinctus* abundance and habitat preferences, herbarium vouchers and iNaturalist occurrences. Coordinates are not provided due to the species’ global conservation status of Critically Endangered. Approximate localities are given in Fig. 1. Leaf samples for genetic analysis are stored in the QUSF Herbarium reference collection in the custody of GR-T.**

Site 1. Original collections (1977–1985)

Dates: 11 August 1977, 17 August 1978, 4 October 1981, 7 July 1985

Elevation: 600 m

Site description: The exact localities of the earliest collections of *G.extinctus* are not known. The type specimen label describes the location as: “Los Ríos or Pichincha: Montañas de Ila, cloud forest along ridge line near La Centinela, Km 12, on road from Patricia Pilar to Flor de Mayo, 600 m.” We believe that these collections were made on the ridge above and continuing north from the community of Centinela del Pichincha, Santo Domingo de los Tsáchilas Province, which is 12 km from Patricia Pilar (Fig. 1). We do not know where on the ridge the collections were made, nor if they were made at one or multiple locations. We did not explore this ridge during the 2021 fieldwork.

Vouchers: *Calaway H. Dodson 11595* (holotype), *Calaway H. Dodson & T. A. Dodson 6809*, *Calaway H. Dodson & T. A. Dodson 15867*, *Calaway H. Dodson, T.A. Dodson & Alvin Embree 7117*

Site 2.

Date: 13 November 2021

Elevation: 659 m

Distance from Site 1: 4.5 km

Site description: This site was located near the top of the road to the Corporación Nacional de Telecomunicaciones antennas at the highest part of the Centinela Range, in a neighbourhood known to local landowners as Bijagual. We searched a fragment of forest along a high ridgeline directly east of the road and did not find *Gasteranthusextinctus* there. We then descended a steep meadow to the south of that fragment until reaching a small creek running west to east. The creek was bordered by a thin strip of old and secondary forest, with cattle pasture to either side. We found three flowering individuals of *G.extinctus* in the pasture – two to the south and one to the north – and one flowering individual in the dark understorey of the creek forest.

Vouchers: No herbarium voucher was collected due to the limited number of individuals; a silica-dried leaf sample is stored at QUSF; https://www.inaturalist.org/observations/101229701, https://www.inaturalist.org/observations/103371741

Site 3.

Date: 13 November 2021

Elevation: 520 m

Distance from Site 1: 6.6 km

Site description: This fragment of relatively well-preserved forest is close to the hamlet of San Pedro de Pambil and mainly surrounded by *Gmelinaarborea* plantations. Two close, but distinct populations were seen at this site: the first with several individuals (10–15) alongside a stream and the second higher up the slope with fewer individuals (3–4).

Vouchers: *Thomas Couvreur et al. 1502* (to be deposited at P, QCA, SEL [078639], WAG, US)

Site 4.

Date: 14 November 2021

Elevation: 620 m

Distance from Site 1: 6.5 km

Site description: This site, which is ~ 1 km from Site 3, is surrounded by a narrow strip of secondary forest that transitions into a *Gmelinaarborea* plantation. The fragment of relatively well-preserved forest is characterised by numerous steep ravines and small streams. *G.extinctus* was seen growing on walls of bare soil above the streambeds and also on flatter parts of the secondary forest.

Vouchers: *Nigel Pitman et al. 11201*, *Nigel Pitman et al. 11202*, *Riley Fortier et al. 210* (to be deposited at F, GUAY, QCNE, SEL, US), https://www.inaturalist.org/observations/101761531

Site 5. Bosque y Cascadas Las Rocas

Date: 15 November 2021

Elevation: 665 m

Distance from Site 1: 19.3 km

Site description: Bosque y Cascadas Las Rocas Private Conservation Area is a privately owned Reserve that protects a primary, humid cloud forest remnant along the Bolo River, near the town of Polanco. We observed *G.extinctus* growing in several clustered populations. One population was adjacent to the main trail, growing on a steep embankment in rich soil with no visible rocks. The other population was growing on almost bare rock next to a waterfall, where it likely receives mist from the falls for most of the day. Another individual was observed on a cliff face adjacent to a tall waterfall.

Vouchers: *Dawson White et al. 830* (to be deposited at QCNE, US),https://www.inaturalist.org/observations/103420808, https://www.inaturalist.org/observations/103420715

Site 6. Cascadas de Cristal

Date: 30 November 2019

Elevation: 990 m (estimated from imagery)

Distance from Site 1: 24.8 km

Site description: Cascadas de Cristal is a privately owned Reserve with a protected forest remnant near the town of Los Ángeles, above the Río Otongo. Observations of *G.extinctus* at this site were made by Josselyn Lizbeth Chacón Ibarra, María Gabriela Sánchez Nicolalde and Marianela Frias in November 2019. Various plants were observed in the forest understorey along the trail to the waterfalls.

Vouchers: https://www.inaturalist.org/observations/36478271, https://www.inaturalist.org/observations/36473651, https://www.inaturalist.org/observatio ns/36415279

Notes on the toponymy of Centinela and Montañas de Ila:

﻿During our visit, we learned that neither of the two names historically used by plant collectors to describe this region are commonly used by local residents. The name ‘Centinela’ is locally used to refer to the small settlement of Centinela del Pichincha, a 12-km drive east of Patricia Pilar, which Dodson and Gentry appear to have used as a base from which to climb up to what they called ‘Centinela Ridge’ or ‘La Centinela’ (see Site 1 above and in Fig. 1). The name ‘Montañas de Ila’ was not familiar to anyone we spoke with during our fieldwork; residents typically referred to individual settlements and neighbourhoods. Until a more precise name can be determined for the broader mountain range, we use ‘the Centinela Region’ to describe the ~ 40-km^2^ landscape above 500 m elevation (the polygons outlined in purple in Fig. 1).
